# Serum Levels of Acylcarnitines and Amino Acids Are Associated with Liberation from Organ Support in Patients with Septic Shock

**DOI:** 10.3390/jcm11030627

**Published:** 2022-01-26

**Authors:** Theodore S. Jennaro, Elizabeth M. Viglianti, Nicholas E. Ingraham, Alan E. Jones, Kathleen A. Stringer, Michael A. Puskarich

**Affiliations:** 1 Department of Clinical Pharmacy and the NMR Metabolomics Laboratory, College of Pharmacy, University of Michigan, Ann Arbor, MI 48109, USA; tjennaro@med.umich.edu (T.S.J.); stringek@med.umich.edu (K.A.S.); 2 Division of Pulmonary and Critical Care Medicine, Department of Internal Medicine, School of Medicine, University of Michigan, Ann Arbor, MI 48109, USA; eviglian@med.umich.edu; 3 Division of Pulmonary, Allergy, Critical Care, and Sleep Medicine, Department of Internal Medicine, School of Medicine, University of Minnesota, Minneapolis, MN 55455, USA; ingra107@umn.edu; 4 Department of Emergency Medicine, University of Mississippi Medical Center, Jackson, MS 39216, USA; aejones@umc.edu; 5 Michigan Center for Integrative Research in Critical Care (MCIRCC), University of Michigan, Ann Arbor, MI 48109, USA; 6 Department of Emergency Medicine, School of Medicine, University of Minnesota, Minneapolis, MN 55415, USA; 7 Department of Emergency Medicine, Hennepin County Medical Center, Minneapolis, MN 55415, USA

**Keywords:** metabolomics, organ failure, sepsis, nuclear magnetic resonance spectroscopy, acylcarnitines, acetylcarnitine, liquid chromatography-mass spectrometry

## Abstract

Sepsis-induced metabolic dysfunction is associated with mortality, but the signatures that differentiate variable clinical outcomes among survivors are unknown. Our aim was to determine the relationship between host metabolism and chronic critical illness (CCI) in patients with septic shock. We analyzed metabolomics data from mechanically ventilated patients with vasopressor-dependent septic shock from the placebo arm of a recently completed clinical trial. Baseline serum metabolites were measured by liquid chromatography-mass spectrometry and ^1^H-nuclear magnetic resonance. We conducted a time-to-event analysis censored at 28 days. Specifically, we determined the relationship between metabolites and time to extubation and freedom from vasopressors using a competing risk survival model, with death as a competing risk. We also compared metabolite concentrations between CCI patients, defined as intensive care unit level of care ≥ 14 days, and those with rapid recovery. Elevations in two acylcarnitines and four amino acids were related to the freedom from organ support (subdistributional hazard ratio < 1 and false discovery rate < 0.05). Proline, glycine, glutamine, and methionine were also elevated in patients who developed CCI. Our work highlights the need for further testing of metabolomics to identify patients at risk of CCI and to elucidate potential mechanisms that contribute to its etiology.

## 1. Introduction

Sepsis is a prevalent, costly, and life-threatening syndrome, formally defined as organ dysfunction occurring secondary to an infection resulting from a dysregulated host response [1,2,3]. Mortality from the most severe form of sepsis, septic shock, approaches 40% [4]. Furthermore, survivors of sepsis experience highly variable clinical trajectories [5], with some patients rapidly recovering (within days) and others developing chronic critical illness (CCI) and suffering profound morbidity, long-term sequela, and an increased risk of late mortality [6,7,8]. The poor outcomes of this latter phenotype are driven in part by the initial sepsis-induced organ injury and dependence on mechanical ventilation or other organ support measures [9]. Furthermore, these patients also have prolonged stays in the intensive care unit (ICU), characterized by cascading, late-onset organ failures [10]. Improved understanding of the patient risk-factors and biologic mechanisms driving CCI are key for the development of novel pharmacotherapy and improvement of long-term sepsis outcomes.

Previous attempts to discriminate who is at risk for developing CCI using electronic health record data at ICU admission have proven unsuccessful [10,11,12]. These findings imply a need for deeper phenotyping of patients, derived from biologic signals of the host response to infection, for full risk stratification and identification of modifiable drug targets. Recent work provides evidence of biologic differences among patients who develop CCI at the transcription, protein, and metabolism levels [13,14,15,16], though further studies in different patient cohorts that leverage and integrate other ‘omic’-technologies are still needed.

Sepsis induces a deranged energy metabolism that manifests as elevated blood lactate and glucose levels, muscle catabolism and perturbed amino acid concentrations, and mitochondrial dysfunction [17,18,19]. Metabolomics, as an applied science, identifies small molecules in a biological sample [20]. In doing so, metabolomics provides a physiological “snapshot” and molecular phenotype of the host and has proven useful in differentiating patient outcomes and response to drug therapy in patients with sepsis [21,22,23,24,25,26,27]. As such, we sought to determine among mechanically ventilated patients with septic shock, if the baseline patient metabolic status could help distinguish CCI from patients with rapid recovery.

## 2. Materials and Methods

### 2.1. Patient Population

**Figure 1:**
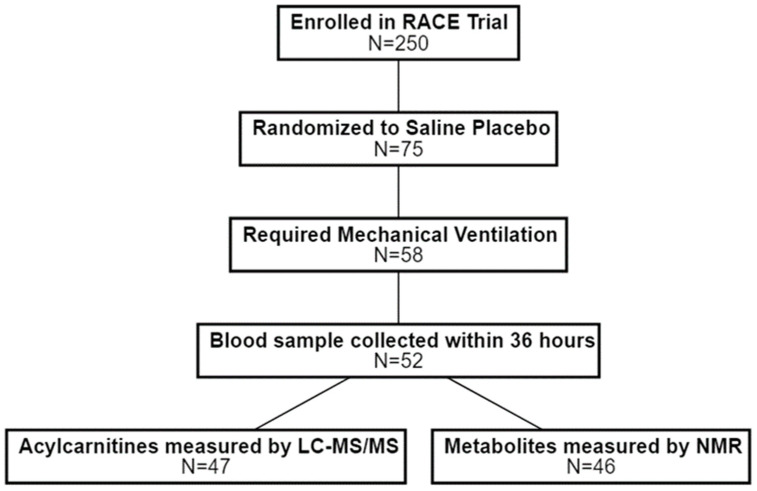
Study flow diagram. Patients were considered for this secondary analysis of the RACE clinical trial if they were randomized to (1) receive saline placebo; (2) required mechanical ventilation; and (3) had a blood sample collected within 36 h of the initiation of mechanical ventilation. Metabolomics data were generated and available for a subset of patients. RACE = Rapid Administration of Carnitine in Sepsis; LC-MS/MS = liquid chromatography mass spectrometry; NMR = nuclear magnetic resonance.

The Effect of Levocarnitine vs. Placebo as an Adjunctive Treatment for Septic Shock —the Rapid Administration of Carnitine in Sepsis (RACE) clinical trial was a multicenter, placebo-controlled, phase II study that adaptively randomized patients with vasopressor-dependent septic shock and moderate organ dysfunction to saline placebo or low (6 g), medium (12 g), or high dose (18 g) levocarnitine [28]. Enrolled patients included adults with a confirmed or suspected bloodstream infection who were identified within 24 h of recognized septic shock and initiation of standardized sepsis treatment guidelines. In addition, inclusion required a total sequential organ failure assessment (SOFA) score [29] greater than 6, a clinical lactate level greater than 2 mmol/L, and treatment with high-dose vasopressors within 4 h of enrollment. Additional inclusion criteria for this secondary analysis were: (a) allocation to the saline placebo treatment arm; (b) receipt of invasive mechanical ventilation; and (c) a serum blood sample available for metabolomics collected within 36 hours of onset of mechanical ventilation (Figure 1). These criteria were chosen to provide a more homogeneous cohort that required multiple types of organ support (both endotracheal intubation and exogeneous vasopressors) and were at high risk of developing CCI. The inclusion of only placebo-treated patients removed any potential modifying effect from a putative metabolic treatment (L-carnitine).

All patients or their legally authorized representative provided informed consent and the trial protocol was registered with clinicaltrials.gov (NCT01665092, accessed on 1 December 2021) and approved by the Institutional Review Board of all participating study sites. The trial was conducted ethically according to Good Clinical Practice guidelines and in accordance with local and federal guidelines and statutes.

### 2.2. Blood Sampling and Metabolomics

Detailed descriptions of the blood sampling, handling, and processing from this cohort have been previously reported [26,30]. Briefly, baseline whole blood samples were collected at the time of clinical trial enrollment. Samples were allowed to clot at room temperature for at least 30 min, aliquoted, and centrifuged to obtain serum. Technical replicates were frozen (−80 °C), de-identified, and shipped on dry ice to the NMR Metabolomics Laboratory at the University of Michigan.

Acylcarnitines were measured by reverse-phase liquid chromatography mass spectrometry (LC-MS/MS) at the Michigan Regional Comprehensive Metabolomics Research Core as previously described [25]. Samples were analyzed using an Agilent 1200 LC coupled to an Agilent 6410 tandem quadrupole (Santa Clara, CA, USA). Absolute quantification using stable isotope internal standards was completed for the following carnitine species: Levocarnitine, C2, C3, C4, C5, C8, C14, C16. Relative quantification by peak area was utilized for 16 additional acylcarnitine compounds.

Abundant polar compounds were measured by quantitative proton nuclear magnetic resonance (^1^H-NMR) on a Varian (now Agilent, Inc., Santa Clara, CA, USA) 11.74 Tesla (500 MHz) spectrometer consistent with our prior methods [31,32]. Spectra were processed using Chenomx NMR Suite 8.2 (Edmonton, AB, Canada) software as previously described [32]. Briefly, compounds were identified in the profile module using the Chenomx spectral library and quantified relative to the area of a formate internal standard (50 µL of 9.64 mM). The complete list of acylcarnitines and NMR metabolites is available in the online supplement (Supplementary Table S1).

In preparation for downstream statistical analysis, missing concentration data in the NMR dataset were assumed to be left-censored and missing not at random due to falling below the limit of detection [33]. As such, missing data were imputed for each metabolite as the minimum concentration observed divided by 2. There were no missing data present in the acylcarnitine dataset. After imputation, metabolite concentrations were log-transformed and standardized to have a mean of zero and standard deviation of one [34,35].

### 2.3. Clinical Outcomes and Statistical Analysis

**Figure 2:**
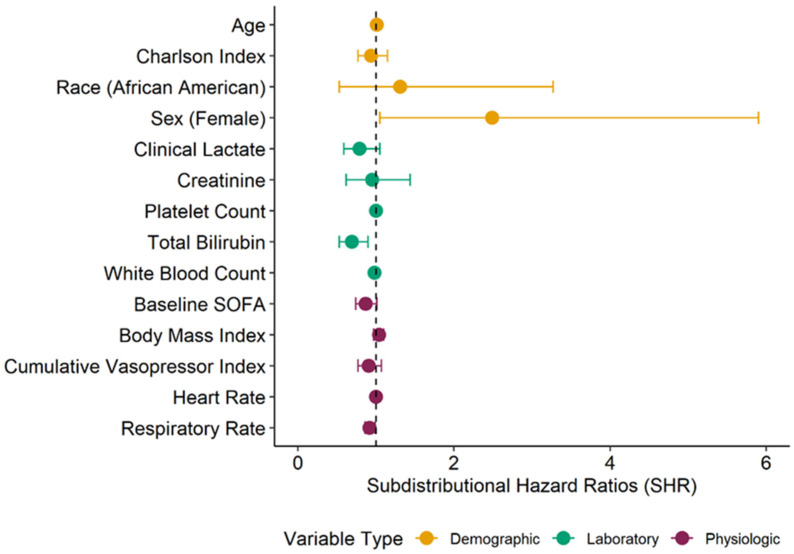
Comparison of time to successful extubation and freedom from vasopressors based on patient characteristics at baseline. The unadjusted subdistributional hazard ratio (SHR) was determined for demographic, clinical laboratory, and physiologic characteristics of patients at time of enrollment. The SHR was determined using a competing risk survival model for time to extubation and freedom from vasopressors, with death in the first 28 days as a competing risk. Here, an SHR < 1 indicates that, with increases in the predictor variable, there is a lower incidence of intact extubation and freedom from vasopressors. Female sex and African American self-reported race were coded as 1, while male sex and Caucasian race were coded as 0. Complete data (N = 47) were available for all variables except race (N = 46); clinical lactate (N = 37); platelet count and cumulative vasopressor index (N = 46); and white blood count (N = 34). Patient characteristics can be found in Supplementary Table S2.

For the primary outcome, we considered each patient’s ventilator-free days and vasopressor-free days as a time-to-event analysis censored at 28 days, with death as a competing risk [36,37]. Specifically, an endpoint of successful extubation without continued need of vasopressors was modeled as a function of time, with death over 28 days considered the competing event. We then fit a series of competing risk survival models, first for patient characteristics at enrollment and then one for each metabolite measured and determined the subdistributional hazard ratio (SHR). Patient characteristics included demographics, clinical laboratory values, and other physiologic parameters (Figure 2). Covariates were chosen a priori, and we adjusted metabolite models for sex, baseline SOFA score, and a modified Charlson Comorbidity Index (see methods in the online supplement), which also accounts for patient age [29,38]. Given the use of sedation in this patient population and its impact on the Glasgow Coma Score, we excluded the neurological component of the SOFA score [39,40]. The *p*-value corresponding to each metabolite’s adjusted SHR was corrected for multiple comparisons according to the false discovery rate (FDR) procedure of Benjamini–Hochberg [41]. Metabolites with an FDR < 0.05 were then rank ordered by the adjusted SHR and plotted with the 95% confidence interval.

To help further visualize the results of the competing risk models, we dichotomized the cohort based on the median value of the top metabolic predictor. We then plotted time to event curves for successful extubation and freedom from vasopressors over 28 days in patients at or above and those below the median metabolite concentration. Patients who died during the 28 days were considered to have zero ventilator-free days and vasopressor-free days to account for the competing risk of death [42].

As an exploratory analysis, we sought to determine the relationship between host metabolism and CCI. Importantly, enrollment and blood sampling in the RACE clinical trial were anchored to onset of septic shock rather than ICU admission. While total patient days in the ICU were recorded, it was not possible to determine how long a patient had been in the ICU prior to enrollment in the study. This complicated our ability to classify patients into common phenotypes of CCI that are derived from ICU length of stay [5,43,44,45]. As such, we classified patients based on a competing risk of death and the continued need for vasopressors and/or mechanical ventilation. Patients were classified as follows: (a) ‘Death’: mortality within 28 days of enrollment; (b) ‘CCI’: survival at 28 days with a continued need for mechanical ventilation and/or vasopressors for at least 14 days; and (c) ‘Rapid Recovery’: survival at 28 days and free from mechanical ventilation and vasopressors before 14 days. We then used Metaboanalyst to perform principal component analysis (PCA) and conducted a one-way ANOVA for each metabolite to determine if there were metabolic differences stratified across outcomes [46]. The ANOVA *p*-values were corrected for multiple comparison as described above, and post-hoc testing for between-group differences was performed for metabolites with an FDR < 0.05 according to Fisher’s Least Square Difference. All data analysis and figure generation were completed in Metaboanalyst (v 5.0; https://www.metaboanalyst.ca/, accessed on 11 November 2021) or RStudio with R (version 3.6.2; Boston, MA, USA) [46,47].

## 3. Results

### 3.1. Patient Characteristics and Time to Freedom from Organ Support

A total of 52 patients from the RACE trial were randomized to the placebo arm and had a baseline blood sample taken within 36 h of the onset of mechanical ventilation. Acylcarnitines data generated by LC-MS/MS were available for 47 patients and corresponding data generated by NMR were available for all but one patient (Figure 1). Of these, 21 patients (*N* = 21/47; 44.7%) were successfully extubated and free of vasopressors over 28 days, 6 (*N* = 6/47; 12.8%) required persistent mechanical ventilation and/or vasopressors, and 20 (*N* = 20/47; 42.6%) died prior to 28 days. Patient characteristics at baseline are provided in the supplement (Supplementary Table S2).

First, we compared the impact of patient characteristics on time to successful extubation and freedom from vasopressors, with any death over 28 days as a competing risk (Figure 2). Female sex was associated with a higher rate of intact extubation and freedom from vasopressors (SHR: 2.49, 95% CI: 1.05–5.90), while respiratory rate (SHR: 0.92, 95% CI: 0.86–0.99) and baseline total bilirubin (SHR: 0.69, 95% CI: 0.53–0.9) were associated with a lower likelihood. In addition, baseline SOFA score (SHR: 0.87, 95% CI: 0.74–1.01) and clinical lactate levels (SHR: 0.79, 95% CI: 0.59–1.05) were moderately related to time to successful extubation. Patient characteristics were otherwise similar based on the primary outcome.

### 3.2. Metabolite Concentrations and Time to Freedom from Organ Support

We identified and measured twenty-four acylcarnitine species by LC-MS/MS (*N* = 47) and 27 small, polar molecules by NMR (*N* = 46). Levocarnitine (LC) and acetylcarnitine (C2) are measured by both methods. A comprehensive list of these metabolites is available in the supplement (Supplementary Table S1). All metabolomics data are publicly available through the National Institutes of Health Metabolomics Workbench (https://www.metabolomicsworkbench.org/; accession number ST001319, accessed on 1 December 2021).

**Figure 3:**
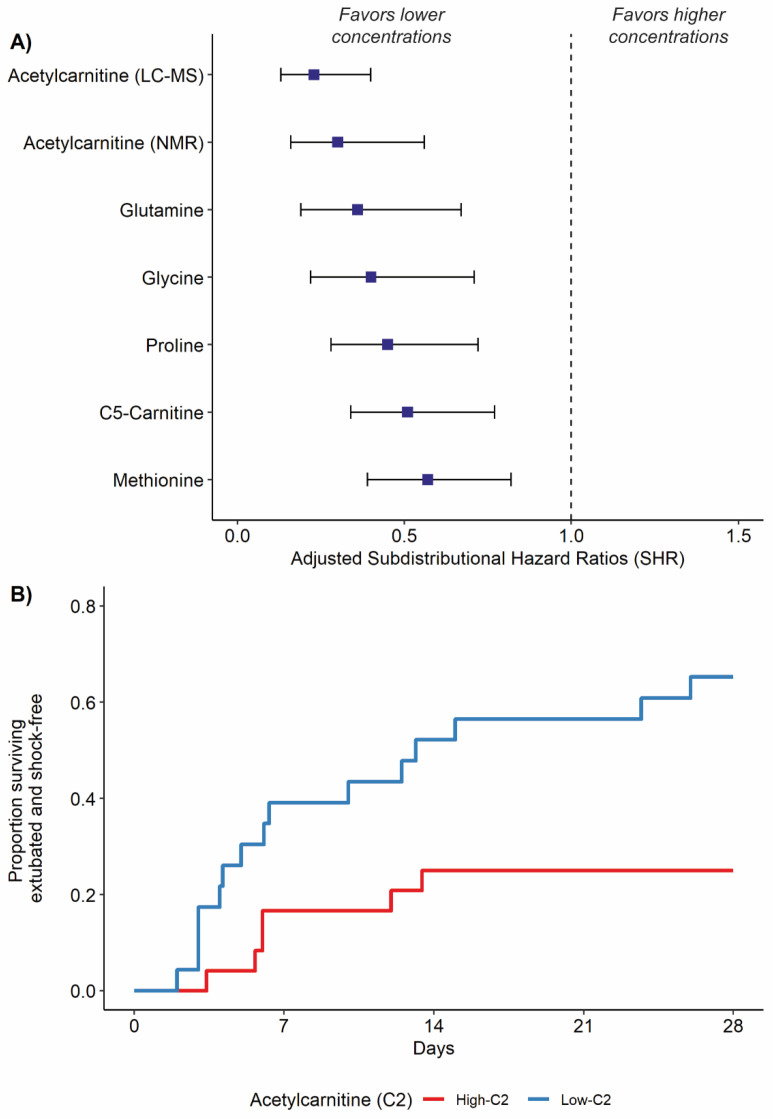
Comparison of time to successful extubation and freedom from vasopressors based on baseline serum metabolite levels. (**A**) The adjusted sub-distributional hazard ratio (SHR) for top metabolic features (FDR < 0.05) related to time to extubation and freedom from vasopressors. The SHR was determined using a competing risk survival model for time to extubation, with death in the first 28 days as a competing risk. Each model was adjusted for baseline SOFA score, sex, and the Charlson comorbidity index. For all metabolites displayed above, lower concentrations were associated with a greater incidence of successful extubation and freedom from vasopressors. (**B**) Visualization of time to breathing unassisted upon dichotomizing the top metabolic feature, acetylcarnitine (C2), above and below the median value. There was a higher proportion of patients with low C2 that survived, were extubated and shock-free over time versus patients with high C2.

In adjusted competing risk survival models, a significant difference was detected in the incidence of successful extubation and freedom from vasopressors based on the baseline concentration of six metabolites (Figure 3A, FDR < 0.05). These metabolic features included acetylcarnitine (C2), valerylcarnitine (C5-carnitine), and four amino acids (glutamine, glycine, proline, and methionine). All significant features had an adjusted SHR less than 1, indicating that elevations in the baseline metabolite concentration were associated with a reduction in the incidence of the event, in this case no longer requiring ICU-level of care [48]. To help further visualize the results of the competing risk models, we stratified patients based on the top metabolic signature, acetylcarnitine (adjusted SHR: 0.23, 95% CI: 0.13–0.40), as measured by LC-MS/MS (Figure 3A). Patients with acetylcarnitine concentrations at or above the median were designated as ‘High-C2’ and those below the median as ‘Low-C2’ (Figure 3B). As a dichotomous variable, the adjusted SHR for acetylcarnitine was 0.28 (95% CI: 0.11–0.75).

### 3.3. Metabolic Differences between CCI and Rapid Recovery with Death as a Competing Risk

**Figure 4:**
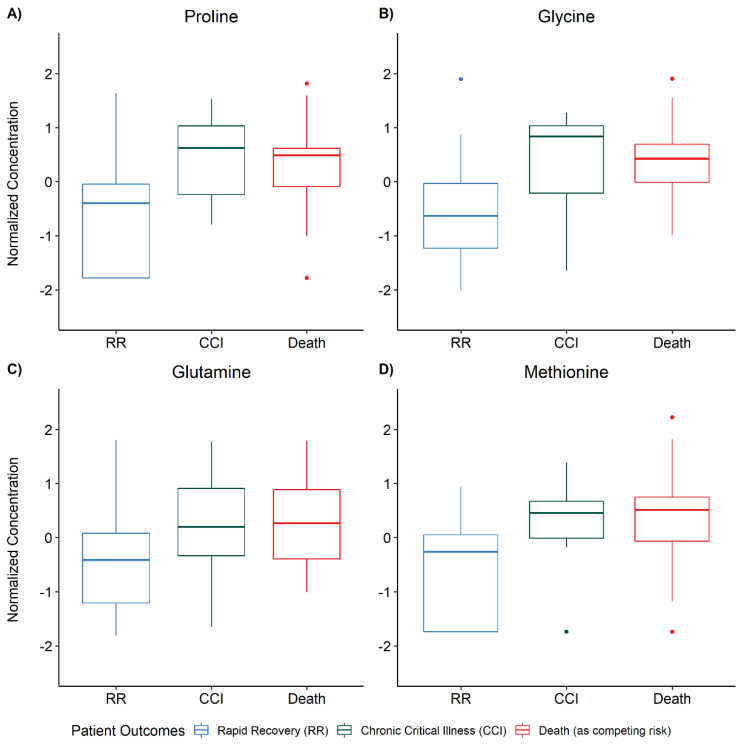
Serum amino acid concentration differences between chronic critical illness (CCI) and rapid recovery (RR) patients. One-way analysis of variance (ANOVA) was used to determine differences in metabolite concentrations stratified by patient outcomes. The ANOVA *p*-values were corrected for multiple comparisons according to the false discovery rate (FDR) procedure of Benjamini–Hochberg and post-hoc testing for between-group differences was done according to Fisher’s Least Square Difference when the FDR was less than 0.05. Four metabolites (proline, glycine, glutamine, and methionine) were different (FDR < 0.05) between patients who developed CCI and those who experienced a RR.

In the exploratory analysis, among patients with both acylcarnitine and NMR data, 9 patients developed CCI (*N* = 9/46; 19.6%), 17 (*N* = 17/46; 37.0%) experienced a rapid recovery, and 20 (*N* = 20/46; 43.5%) died prior to 28 days. Patients who experienced a rapid recovery were more likely to be alive at one year compared to patients who developed CCI (82.4% vs. 66.7%, *p* = 0.03). In multivariable PCA analysis, metabolic differences were most pronounced in the mortality outcome group. There was substantial overlap between patients who developed CCI and those who experienced rapid recovery (Supplemental Figure S1). In our univariate one-way ANOVA analysis, 24 metabolites were significantly different among the three groups (ANOVA FDR < 0.05). Post-hoc testing for between-group differences by Fisher’s Least Square Differences revealed this was largely driven by metabolic differences in the mortality outcome group (Supplementary Table S3). Nonetheless, after post-hoc testing, the same four amino acids identified in our organ failure support analysis were also elevated in patients who went on to develop CCI relative to those who had a rapid recovery (FDR < 0.05, Figure 4, Supplementary Table S3).

## 4. Discussion

Our study sought to determine if metabolic differences among mechanically ventilated patients with septic shock were associated with the liberation from organ support and duration of ICU-level of care. In a competing risk, time-to-event analysis we demonstrated that serum concentrations of short chain acylcarnitines (C2 and C5) and four amino acids (proline, glycine, glutamine, and methionine) are related to liberation from mechanical ventilation and vasopressors over 28 days. Additionally, we found that these same amino acids were elevated in CCI patients who required at least 14 days of mechanical ventilation and/or vasopressors relative to those who rapidly recovered. Our findings provide new insights into candidate biochemical pathways that are perturbed in sepsis survivors and suggest metabolomics may provide prognostic detail beyond mortality outcomes.

A dysregulated host metabolic response is formally defined in the Sepsis-3 definition and is being increasingly understood as a hallmark of sepsis pathophysiology [1,49]. This perturbation of metabolism has been consistently linked with alterations in energy utilization, mitochondrial dysfunction, organ failure, and mortality [19,24,50,51,52,53]. Our work supports this growing body of evidence, finding that the mortality group in our study was the most metabolically disrupted (Supplementary Figure S1 and Table S3). Perhaps more importantly, however, our study introduces the utility of metabolomics to differentiate sepsis survivor phenotypes, CCI, and rapid recovery. The role of deranged metabolism in CCI following sepsis survival is best understood in work surrounding the persistent inflammation, immunosuppression and catabolism syndrome [54,55,56]. This syndrome is characterized in part by persistent inflammation leading to profound muscle catabolism and a cachexia-like response [5,57]. Elevations in two key metabolites from our study, acetylcarnitine and valerylcarnitine (Figure 3), are broadly indicative of altered energy demand, β-oxidation of fatty acids, mitochondrial dysfunction, and metabolic inflexibility [58]; and acetylcarnitine was recently further linked with the systemic inflammatory response in patients with sepsis [52]. Here, we implicate short-chain acylcarnitines as markers of not only mortality, but also the differential need for life-supporting measures in the ICU.

Other proposed metabolic biomarkers of CCI have included low serum albumin and increased frailty (as a surrogate for poor nutritional status) [59], and the urea to creatinine ratio, a biochemical signature related to muscle catabolism [15,60]. We were not able to assess the impact of serum albumin or measures of frailty in our cohort, and although creatine, a key metabolite of skeletal muscle energy homeostasis, was detected by our NMR assay, it was not found to be strongly related to the time to extubation and freedom from vasopressors. However, serum concentrations of four amino acids (all of which are non-essential except for methionine) were strongly related in both our competing risk models and when CCI was defined with a 14-day cutoff point.

Differentiating blood levels of amino acids are metrics of the overall energy economy of the host and are related to patient outcomes in critical illnesses [61,62]. We have previously shown that serum levels of methionine are increased in patients with persistent septic shock compared with those whose shock resolved [63]. Methionine is important for immune function and its dietary restriction has been shown to decrease inflammation and improve skeletal muscle health in animal models [64,65]. Glutamine is the most abundant amino acid in humans and sources numerous metabolic pathways, many of which are important in maintaining energy homeostasis [66]. It and glycine are precursors of the antioxidant glutathione [67], and glutamine, glycine and proline are all precursors of bacterial (microbiome) production of short-chain fatty acids (e.g., butyrate) which participate in maintaining immune function [68]. In critically ill patients, both low [69,70] and high levels [71,72] of glutamine have been previously shown to be related to mortality, and supplementation has failed to consistently demonstrate clinical benefit [73,74]. This has led many to question the indiscriminate supplementation of glutamine [75,76] and suggests a precision approach may be warranted. In this cohort of patients with septic shock, high levels of glutamine were related to a prolonged need for organ support and poor clinical outcomes; further studies in this specific patient population are warranted. In aggregate, our findings suggest that metabolic differences among patients with septic shock may lend insight into mechanisms that contribute to sepsis outcome phenotypes and could be used as predictive biomarkers of CCI. Future metabolomics studies in patients at risk of CCI will permit further assessment of the prognostic value of candidate metabolite biomarkers and inform targeted metabolic pharmacotherapy and/or adjunctive nutritional support.

Our study has important limitations that warrant further consideration. First, our study was cross-sectional in nature, leveraging only a single metabolic timepoint. While the collection of serum samples was carefully anchored to a clinical event (onset of mechanical ventilation), future work that follows the trajectory of metabolic changes in the ICU may provide additional prognostic value and mechanistic insight. In addition, we considered only a limited read of the serum metabolome using normalized concentrations and acknowledge that our metabolomics data are not comprehensive. Absolute quantification of potential biomarkers will be essential for ultimate clinical translation, while a broader read of the metabolome combined with data acquired at the transcription and protein level offers an exciting and potentially more fruitful assessment of the pathophysiology of CCI. Moreover, we used a definition of CCI based on the continuous need for mechanical ventilation and vasopressors, while much of the CCI literature relies on ICU length of stay. Finally, our study was observational and thus our findings are hypothesis-generating and require rigorous validation in prospective cohorts.

## 5. Conclusions

Among mechanically ventilated patients with septic shock, serum concentrations of two acylcarnitines and four amino acids were related to the time to extubation and freedom from vasopressors. Our work supports the feasibility of metabolomics to interrogate the mechanisms of CCI and the hypothesis that altered host metabolism is a sign of and/or contributes to CCI.

## Data Availability

All metabolomics data are publicly available through the National Institutes of Health Common Fund at the Metabolomics Workbench (https://www.metabolomicsworkbench.org/; accession number ST001319, accessed on 1 December 2021).

## Supplementary Materials

The following are available online at https://www.mdpi.com/article/10.3390/jcm11030627/s1, Table S1: Metabolites detected and quantified by LC-MS/MS and ^1^H-NMR. Table S2: Patient characteristics. Table S3: One-way analysis of variance (ANOVA) for differences in metabolite concentrations stratified by patient outcomes. Figure S1: Principal component analysis (PCA) stratified by patient outcomes.
