# Supplementary material for: Serum Levels of Acylcarnitines and Amino Acids Are Associated with Liberation from Organ Support in Patients with Septic Shock
Source: J Clin Med. 2022 Jan 26;11(3):627. doi: 10.3390/jcm11030627 (PMC8836990; doi:10.3390/jcm11030627)
Supplement: Supplementary file 1 [file jcm-11-00627-s001.zip › jcm-1514140-supplementary.pdf]

Supplementary Material

Understanding Metabolic Perturbations of Chronic Critical Illness in Mechanically Ventilated Patients with Septic Shock

Theodore S. Jennaro, Elizabeth M. Viglianti, Nicholas E. Ingraham, Alan E. Jones, Kathleen A. Stringer, and Michael A. Puskarich*

Methods:

Definition of modified Charlson Comorbidity Index: Patient demographics, comorbidities, and clinical outcomes were recorded and are maintained in a secure research electronic data capture (REDCap) database [1]. However, it was not possible to obtain all comorbidities necessary to calculate a full comorbidity score according to Charlson [2]. Specifically, we lacked information on peptic ulcer disease and connective tissues diseases and could not distinguish between uncomplicated diabetes vs. diabetes with residual organ damage or HIV positive vs. Acquired immunodeficiency syndrome. Thus, our modified scale assigns a score of +1 to any patient with documented diabetes and does not account for the remaining disease states.

Results:

Supplementary Table S1: **Metabolites detected and quantified by LC-MS/MS and ^{1}H-NMR.** The Kyoto Encyclopedia of Genes and Genomes (KEGG) identification (ID) number is provided when available (+). When a corresponding KEGG ID does not exist, the human metabolomics database (HMDB) ID number is provided (*). When neither a KEGG nor HMDB ID is available, a metabolite is assigned N/A. The missingness for NMR metabolites represents the percentage of samples for which that respective compound did not have a reported value.

**LC-MS/MS Metabolites**		**^{1}H-NMR Metabolites**		
Compound Name	KEGG ID^{+}	Compound Name	KEGG ID^{+}	Missingness
Levocarnitine	C00318	2-Hydroxybutyrate	C05984	0%
Acetylcarnitine (C2)	C02571	2-Oxoisocaproate	C00233	17.4%
Propanoylcarnitine (C3)	C03017	3-Hydroxybutyrate	C01089	2.2%
Butanoylcarnitine (C4)	C02862	Acetylcarnitine (C2)	C02571	2.2%
Valerylcarnitine (C5)	HMDB0013128*	Alanine	C00041	0%
Hexanoylcarnitine (C6)	HMDB0000756*	Betaine	C00719	2.2%
C8:1-carnitine	HMDB0013324*	Levocarnitine	C00318	10.9%
Octanoylcarnitine (C8)	C02838	Choline	C00114	19.6%
C10:1-carnitine	HMDB0240585*	Citrate	C00158	15.2%
Decanoylcarnitine (C10)	C03299	Creatine	C00300	0%
C12:1-carnitine	HMDB0013326*	Creatinine	C00791	0%
Dodecanoylcarnitine (C12)	HMDB0002250*	Glucose	C00221	0%
C14:1-carnitine	HMDB0002014*	Glutamine	C00064	0%
Tetradecanoylcarnitine (C14)	HMDB0005066*	Glycine	C00037	0%
C16:1-carnitine	HMDB0006317*	Histidine	C00135	8.7%
C20:4-carnitine	HMDB0006455*	Isoleucine	C00407	15.2%
C18:2-carnitine	HMDB0006469*	Lactate	C00186	0%
C20:3-carnitine	N/A	Leucine	C00123	0%
Palmitoylcarnitine (C16)	C02990	Lysine	C00047	2.2%
C18:1-carnitine	HMDB0006464*	Methionine	C00073	15.2%
C20:2-carnitine	N/A	Ornithine	C00077	26.1%
Stearoylcarnitine (C18)	HMDB0000848*	Phenylalanine	C00079	0%
C20:1-carnitine	N/A	Proline	C00148	15.2%
Arachidonoylcarnitine (C20)	HMDB0006455*	Propylene glycol	C00583	0%
		Pyruvate	C00022	0%
		Tyrosine	C00082	0%
		Valine	C00183	0%

Supplementary Table S2: **Patient characteristics.**

Patient Characteristic	N = 47
Age (years)[1]	61 (46, 70)
Sex[2]	
Female	21 (45%)
Male	26 (55%)
Race[2]	
African American	14 (30%)
Caucasian	32 (70%)
Unknown	1
Charlson Comorbidity Index[1]	4.00 (2.00, 5.50)
Heart Rate (beats/minute)[1]	103 (94, 113)
Respiratory Rate (breaths/minute)[1]	20.0 (16.5, 25.0)
Cumulative vasopressor index[1]	4.00 (4.00, 8.00)
Unknown	1
Body mass index[1]	27 (22, 35)
SOFA score[1,3]	9.00 (8.00, 11.00)
White blood count (cells/mm^3)[1]	24 (12, 31)
Unknown	13
Platelet (cells/mm^3)[1]	147 (126, 214)
Unknown	1
Creatinine (mg/dL)[1]	1.60 (1.16, 2.33)
Total bilirubin (mg/dL)[1]	1.30 (0.60, 3.30)
Clinical lactate (mmol/L)[1]	3.60 (2.30, 6.50)
Unknown	10

[1]Median (IQR)
[2]N (%)
[3]Neurological component removed

Supplementary Table S3: One-way analysis of variance (ANOVA) for differences in metabolite concentrations stratified by patient outcomes. The ANOVA p-values were corrected for multiple comparisons according to the false discovery rate (FDR) procedure of Benjamini–Hochberg. For metabolites with an FDR <0.05, post-hoc testing for between group differences was done according to Fisher's Least Square Difference and reported when significant (FDR <0.05). CCI = chronic critical illness; RR = rapid recovery.

Metabolite	F-Value	P-Value	FDR	Post-hoc Testing
C16:1-carnitine	14.38	1.65E-05	8.43E-04	Death vs. CCI; Death vs. RR
C14:1-carnitine	12.36	5.75E-05	1.47E-03	Death vs. CCI; Death vs. RR
Dodecanoylcarnitine (C12)	11.56	9.61E-05	1.63E-03	Death vs. CCI; Death vs. RR
Tetradecanoylcarnitine (C14)	10.95	1.43E-04	1.83E-03	Death vs. CCI; Death vs. RR
Palmitoylcarnitine (C16)	10.20	2.38E-04	2.42E-03	Death vs. CCI; Death vs. RR
Acetylcarnitine (C2, LC-MS)	9.69	3.35E-04	2.82E-03	Death vs. CCI; Death vs. RR
Decanoylcarnitine (C10)	9.22	4.66E-04	2.82E-03	Death vs. CCI; Death vs. RR
C12:1-carnitine	9.10	5.05E-04	2.82E-03	Death vs. CCI; Death vs. RR
C20:2-carnitine	9.04	5.29E-04	2.82E-03	Death vs. CCI; Death vs. RR
C18:1-carnitine	8.97	5.53E-04	2.82E-03	Death vs. CCI; Death vs. RR
C18:2-carnitine	8.32	8.79E-04	4.08E-03	Death vs. CCI; Death vs. RR
C20:1-carnitine	7.93	1.17E-03	4.97E-03	Death vs. CCI; Death vs. RR
Acetylcarnitine (C2, NMR)	7.81	1.28E-03	5.02E-03	Death vs. RR
Stearoylcarnitine (C18)	7.06	2.23E-03	8.13E-03	Death vs. CCI; Death vs. RR
C20:3-carnitine	6.62	3.11E-03	1.06E-02	Death vs. CCI; Death vs. RR
Proline	6.41	3.66E-03	1.17E-02	CCI vs. RR; Death vs. RR
C10:1-carnitine	5.68	6.48E-03	1.94E-02	Death vs. CCI; Death vs. RR
Octanoylcarnitine (C8)	5.45	7.80E-03	2.03E-02	Death vs. CCI; Death vs. RR
Glycine	5.43	7.90E-03	2.03E-02	CCI vs. RR; Death vs. RR
C20-carnitine	5.42	7.95E-03	2.03E-02	Death vs. CCI; Death vs. RR
Propanoylcarnitine (C3)	5.05	1.07E-02	2.60E-02	Death vs. RR
Glutamine	4.61	1.53E-02	3.55E-02	CCI vs. RR; Death vs. RR
Methionine	4.32	1.95E-02	4.32E-02	CCI vs. RR; Death vs. RR
C20:4-carnitine	4.11	2.32E-02	4.93E-02	Death vs. CCI
L-Carnitine (LC-MS)	4.03	2.48E-02	5.06E-02	NA
Valerylcarnitine (C5)	3.76	3.13E-02	6.14E-02	NA
L-carnitine (NMR)	3.46	4.05E-02	7.65E-02	NA
Betaine	3.25	4.83E-02	8.81E-02	NA
Propylene Glycol	3.09	5.56E-02	9.77E-02	NA
Alanine	2.94	6.36E-02	1.08E-01	NA
Citrate	2.78	7.31E-02	1.20E-01	NA
3-Hydoxybutyrate	2.55	8.98E-02	1.43E-01	NA
Tyrosine	2.45	9.80E-02	1.52E-01	NA
Phenylalanine	2.34	1.08E-01	1.63E-01	NA
Choline	2.05	1.42E-01	2.06E-01	NA

Leucine	1.87	1.67E-01	2.37E-01	NA
Histidine	1.82	1.75E-01	2.41E-01	NA
2-Hydroxybutyrate	1.69	1.97E-01	2.58E-01	NA
Butanoylcarnitine (C4)	1.65	2.03E-01	2.58E-01	NA
Hexanoylcarnitine (C6)	1.65	2.04E-01	2.58E-01	NA
Lysine	1.63	2.07E-01	2.58E-01	NA
Valine	1.50	2.34E-01	2.84E-01	NA
Lactate	1.38	2.62E-01	3.10E-01	NA
C8:1-carnitine	1.05	3.60E-01	4.17E-01	NA
Glucose	0.81	4.52E-01	5.12E-01	NA
Isoleucine	0.77	4.70E-01	5.21E-01	NA
Creatine	0.70	5.02E-01	5.45E-01	NA
Pyruvate	0.60	5.53E-01	5.88E-01	NA
Ornithine	0.25	7.78E-01	8.09E-01	NA
2-Oxoisocaproate	0.12	8.89E-01	9.07E-01	NA
Creatinine	0.07	9.32E-01	9.32E-01	NA

Supplementary Figure S1. **Principal component analysis (PCA) stratified by patient outcomes.** The first three principal components explained ~62% variation; 33.9% (PC1), 18.4% (PC2), and 9.3% (PC3). Patients who required at least 14 days of mechanical ventilation or vasopressors were classified as chronic critical illness (‘CCI’, blue circles) and were more similar to patients who experienced rapid recovery (‘Rapid-Recovery’, purple diamonds) than patients who died at or prior to 28 days (‘Competing-Death’, orange triangles).

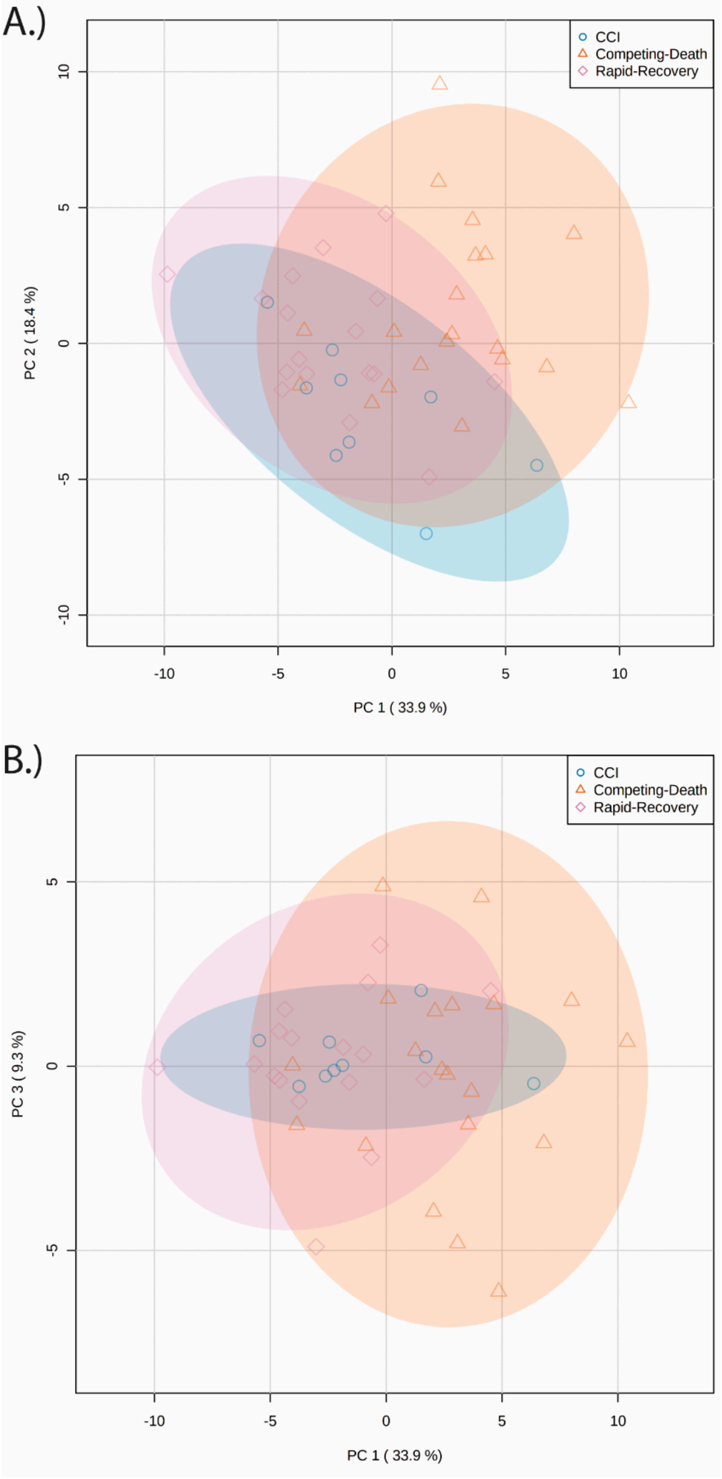

References:

1. Harris PA, Taylor R, Thielke R, Payne J, Gonzalez N, Conde JG. Research electronic data capture (REDCap)--a metadata-driven methodology and workflow process for providing translational research informatics support. J Biomed Inform. 2009;42(2):377-81. Epub 2008/10/22. doi: 10.1016/j.jbi.2008.08.010. PubMed PMID: 18929686; PubMed Central PMCID: PMCPMC2700030.

2. Charlson ME, Pompei P, Ales KL, MacKenzie CR. A new method of classifying prognostic comorbidity in longitudinal studies: development and validation. J Chronic Dis. 1987;40(5):373-83. Epub 1987/01/01. doi: 10.1016/0021-9681(87)90171-8. PubMed PMID: 3558716.
